# Self-assembling peptide hydrogel enables instant epicardial coating of the heart with mesenchymal stromal cells for the treatment of heart failure

**DOI:** 10.1016/j.biomaterials.2017.10.050

**Published:** 2018-02

**Authors:** Yuki Ichihara, Masahiro Kaneko, Kenichi Yamahara, Marinos Koulouroudias, Nobuhiko Sato, Rakesh Uppal, Kenji Yamazaki, Satoshi Saito, Ken Suzuki

**Affiliations:** a William Harvey Research Institute, Barts and the London School of Medicine and Dentistry, Queen Mary University of London, United Kingdom; b Cardiovascular Surgery, Tokyo Women's Medical University, Japan; c Transfusion Medicine and Cellular Therapy, Hyogo College of Medicine, Japan; d Kaneka Corporation, Osaka, Japan

**Keywords:** Self-assembling peptide hydrogel, Bioengineering, Mesenchymal stromal cell, Cell transplantation therapy, Heart failure

## Abstract

Transplantation of mesenchymal stromal cells (MSCs) is an emerging therapy for the treatment of heart failure. However, the delivery method of MSC is currently suboptimal. The use of self-assembling peptide hydrogels, including PuraMatrix^®^ (PM; 3-D Matrix, Ltd), has been reported for clinical hemostasis and in research models. This study demonstrates the feasibility and efficacy of an advanced approach for MSC-therapy, that is coating of the epicardium with the instantly-produced PM hydrogel incorporating MSCs (epicardial PM-MSC therapy). We optimized the conditions/procedure to produce “instant” 2PM-MSC complexes. After spreading on the epicardium by easy pipetting, the PM-MSC complex promptly and stably adhere to the beating heart. Of note, this treatment achieved more extensive improvement of cardiac function, with greater initial retention and survival of donor MSCs, compared to intramyocardial MSC injection in rat heart failure models. This enhanced efficacy was underpinned by amplified myocardial upregulation of a group of tissue repair-related genes, which led to enhanced repair of the damaged myocardium, *i.e.* augmented microvascular formation and reduced interstitial fibrosis. These data suggest a potential for epicardial PM-MSC therapy to be a widely-adopted treatment of heart failure. This approach may also be useful for treating diseases in other organs than the heart.

## 1 Introduction

Transplantation of mesenchymal stromal/stem cells (MSCs) has been developed as a promising new approach for various diseases that are difficult to treat with current treatments, including graft-versus-host disease, autoimmune diseases, and heart failure [1], [2], [3]. In addition to their capacity for anti-inflammation and tissue repair, MSCs have an advantage as a potential donor for stem cell therapy in the utility for allogeneic cell transplantation. Although autologous stem cells are immunologically most appropriate, cells from aged patients with multiple comorbidities have compromised therapeutic efficacy and reduced *in-vitro* expansion ability [4]. Furthermore, the use of autologous stem cells requires an invasive biopsy from the patient and takes protracted culture for expansion, and quality control of each cell product, which imposes significant logistic, economic, and timing constraints [5]. As such, the use of allogeneic MSCs would allow the development of an MSC-bank, enabling an “off-the-shelf” supply of quality-assured MSCs with reduced costs. The most common source for MSCs is bone marrow. However, adipose-tissue, fetal-membrane, amnion membrane, and cord blood are also considered to be promising sources [6], [7], [8], [9].

The potential of MSC transplantation for heart failure has been extensively reported in animal models [10], [11]. While MSCs may not offer clinical benefits via differentiation to cardiomyocytes, they are able to secrete growth factors, cytokines, chemokines, exosomes, and microRNAs, which stimulate intrinsic self-repair systems to favor recovery of viable but failing myocardium [5], [12]. Despite this, clinical trials of MSCs for heart disease to date reported only “modest (if preliminary) effects” [5]. One solution to overcome this limitation may lie in refining the method of cell delivery to the heart. Intramyocardial, intravenous and intracoronary injection techniques are currently used, but all of these methods result in poor donor cell survival [13], [14], [15], [16]. Epicardial placement (not injection) is an alternative route for cell delivery to the heart. We have reported that epicardial placement of MSCs in the form of a “cell-sheet”, produced in temperature-responsive dishes markedly improved donor cell survival and amplified therapeutic effects compared to intramyocardial injection in rat models of acute MI and heart failure [17], [18]. As a result, this technique enhanced repair of the damaged myocardium in association with amplified upregulation of reparative factors and augmented cardiac function compared to intramyocardial injection. Furthermore, epicardial placement is free from the risks of coronary embolism and arrhythmogenesis [14], [15], [16], [17]. Other reports demonstrated the efficacy of alternative epicardial placement methods, including the use of fibrin glue or pre-made tissue-engineered contracts [19], [20].

Self-assembling peptide hydrogels may have the potential to achieve more effective epicardial placement of MSCs on the heart. On exposure to salt, this fully-synthetic material assembles into nanofibers on a scale similar to the extracellular matrix, forming a histocompatible and bioresorbable hydrogel. PuraMatrix^®^ (PM; 3-D Matrix, Ltd.) is one of the most extensively studied among this type of hydrogel. PM consists of 99% water and amino acids (1% w/v; sequences of Arginine-Alanine-Aspartic Acid-Alanine) [21], [22]. Under physiological conditions, the peptide component of PM self-assembles into a 3-dimensional hydrogel that exhibits a highly organized nanometer scale fibrous structure with an average pore size of 50–200 nm [22]. The soluble material can be spread onto organs *in-vivo* and will subsequently form a hydrogel. Morphology and rheological properties of PM gel have been already reported in details [22], [23], [24], [25]. In particular, this gel has been suggested to be effective for hemostasis during surgery [26]. We hypothesized that such controllable gelation, adhesiveness, and easy handling characteristics of PM both pre- and post-gelation will realize an advanced method of epicardial placement, that is epicardial “coating” with a PM-MSC complex which is instantly produced in the operating room at the time of surgery. This technique negates the need for not only labor/cost-demanding GMP-production and transportation of cell-sheets or pre-made constructs, but also expensive GMP-cell culture facility in the treatment hospital. Instead, it is proposed that ready-to-use MSCs will be delivered by the hub cell processing center to each hospital. Such an approach should enable the use of this technique by any cardiac surgeon in any hospital.

This study therefore aimed to firstly optimize a protocol to instantly produce the PM-MSC complex that can be used clinically during surgery by applying it directly onto the heart with a simple application procedure. Secondly we aimed to investigate the feasibility and efficacy of the use of the instantly-produced PM-MSC complex in epicardial “coating” of the heart in clinically-relevant animal models.

## 2 Materials and methods

All studies were performed with the approval of the institutional ethics committee and the Home Office, UK. The investigation conforms to the Principles of Laboratory Animal Care formulated by the National Society for Medical Research and the Guide for the Care and Use of Laboratory Animals (US National Institutes of Health Publication, 1996). All in-vivo and in-vitro assessments were carried out in a blinded manner.

### 2.1 Collection of MSCs

#### 2.1.1 Rat fetal membrane-derived MSCs

Fetal membrane-derived mesenchymal stem/stromal cells (MSC) were collected from wild type pregnant Lewis or Sprague-Dawley rats (pregnant day 19–20; purchased from Charles River, UK) and expanded following a reported protocol [27], [28]. Collected cells were placed in 25 cm^2^ flasks (Nunc) with an initial plating concentration of approximately 1 × 10^6^ cells/cm^2^, and cultured in αMEM (Gibco) with 10% inactivated fetal bovine serum (FBS) containing L-glutamine (200 mM; Gibco), penicillin (100 U/ml) and streptomycin (100 mg/ml; Sigma), at 37 °C in a humidified atmosphere containing 95% air/5% CO_2._ (incubator: Binder, Germany). The culture medium was aspirated and changed every 48–72 h without additional washing. When cell confluency reached 80–90%, cells were passaged by detachment using 0.25% Trypsin/0.2% EDTA (Sigma). Plating concentrations for subsequent passages were approximately 1 × 10^4^ cells/cm^2^.

#### 2.1.2 Rat bone marrow-derived MSCs

Bone marrow-derived MSCs were collected from the bone marrow of the tibias and femurs of male Lewis rats (100–150 g; Charles River UK) and expanded as we have described previously [17], [18], [29]. Collected cells were cultured in αMEM with 20% inactivated FBS containing L-glutamine, penicillin and streptomycin under the same conditions as above.

#### 2.1.3 Human amnion-derived MSCs

Human amnion-derived MSCs were collected as previously described [8]. The Ha-MSCs were isolated from fetal membranes of healthy donor mothers after the delivery (cesarean section) with written informed consent obtained. 1 × 10^6^ of cryopreserved Ha-MSCs were defrosted and plated in 75 cm^2^ flasks (Nunc). αMEM with 15% inactivated FBS containing L-glutamine, penicillin and streptomycin was used for cell cultivation under the same conditions as above.

### 2.2 Characterization of MSCs

At each passage, cell numbers and viability were counted by Countess Cell Counter (Invitrogen) with Trypan blue staining and doubling time was estimated.

#### 2.2.1 Cell surface marker detection by flow-cytometric analysis

For cell-surface marker characterization using flow-cytometry, 1 × 10^6^ MSCs were stained with 1:100 dilution of fluorescein isothiocyanate-conjugated anti-CD34 (Santa Cruze, USA), CD45 (Chemicon; Hampshire, UK), CD90 (Abcam, Cambridge, UK) or Alexa 647-conjugated anti-CD29 (Biolegend, London, UK) antibodies. Corresponding isotype-matched control antibodies were used for negative controls. All antibodies were used at 1:100 dilution following instructions stipulated by the company's guidelines. Samples were analyzed using the Dako Cyan flow-cytometer (Dako Cytomation, UK).

#### 2.2.2 Osteogenic and adipogenic differentiation assay

MSCs were plated on 24-well plates and subjected to adipogenic or osteogenic differentiation medium. Adipogenic differentiation medium was α-minimal essential medium (α-MEM) supplemented with 100 μM isobutyl methylxanthine (Sigma-Aldrich, UK), 60 μM indomethacin (Fluka; Dorset, UK), 1 μg/ml insulin (Sigma-Aldrich), and 0.5 μM hydrocortisone (Sigma-Aldrich), while osteogenic differentiation medium was α-MEM supplemented with 0.1 μM dexamethasone (Sigma-Aldrich), 10 mM β-glycerophosphate (Sigma-Aldrich), and 0.05 mM ascorbic acid (Sigma-Aldrich). Medium was changed every 2–3 days. After 3 weeks of incubation, cells were fixed with 4% paraformaldehyde, and stained with Oil red O (Fluka) for detecting adipocytes containing lipid vacuoles or with Alizarin red (Fluka) to detect osteocytes containing calcium deposits.

### 2.3 Production and characterization of the PM- MSC complex

PM (2.5%; 3D-Matrix, Japan) and MSC suspension were mixed in differing ratios and subject to the following *in vitro* assessments: (1) pH, (2) MSC survival (Propidium Iodide staining), (3) adhesiveness (placement on the plastic plate, which was tilted) and (4) ease of mixture. In addition, the viscoelastic property of PM-MSC complex with different PM doses was assessed using an AR2000 rheometer (TA Instruments, USA), fitted with a 40 mm 4° cone [22]. Immediately after mixture of PM and MSC suspension, the total 800 μl of each mixture was pipetted onto the plate of the rheometer. After 10 min, frequency sweep tests were performed at 1 rad/sec at 1 Pa.

### 2.4 Viability of MSCs in the PM-MSC complex

1 × 10^6^ of each type of MSC pellet was diluted with 100 μl of HBSS (MSC/HBSS suspension), and subsequently resuspended with 100 μl of PM mixture (0.2–2.5%) containing sucrose solution with corresponding concentration. Same number of cells was suspended with 200 μl of PBS as a control (without PM). 5 μl of propidium iodide (PI) solution (Calbiochem, UK) was added to the PM-MSC complex. After 10 and 60 min observation, 10 μl of that was taken and placed on the slide (Thermo Scientific, UK), then enclosed with round type cover glass (VWR, UK). PI-positive cells were detected and analyzed by fluorescence microscopy (BZ8000; Keyence). In addition, cell viability was confirmed by using a Live and Dead Cell Assay kit (Abcam, UK) according to the company's instruction.

### 2.5 Retention of the PM-MSC complex

A total of 200 μl of PM-MSC complex with 1 × 10^6^ cells was prepared as mentioned above. 20 μl of the complex was taken and placed on a drawn circle with a diameter of 10 mm on the top edge of a slide. The slide was then tilted at 90°. After 5-min observation, the length of footage of dropped cell suspension from the circle along the slide was measured and the ratio to total length of slide was calculated.

### 2.6 Neutralization of the PM

To attenuate an acidity of PM, 16 μl of 0.2 N sodium hydroxide (NaOH) was added to 84 μl of the 0.8% PM with sucrose solution, followed by mixture with 100 μl of MSC/HBSS suspension. Separately, to reduce the compacted aggregation formation during the neutralized process of the hydrogel, 60 μl of 7.5% sodium bicarbonate (NaHCO_3_) was added to 100 μl of 1.0% PM mixture containing 20 μl of 25% sucrose solution instead of NaOH. The PM hydrogel was then sonicated with 5–10 kHz for 15 s by probe sonicator (Soniprep 150; MSE, UK) to break down the compacted aggregation formation, followed by centrifugation at 4000 rpm for 5 min. After removal of supernatant, MSC pellet was directly suspended with this modified PM product thoroughly. The pH level of the neutralized modified PM product and cell viability in modified PM-MSC complex 10 min and 60 min after mixture were measured by the method described above. The tilting test with the modified PM-MSC complex was also performed.

### 2.7 In vivo surgical procedure

Myocardial infarction (MI) was induced in male Lewis rats (approximately 200 g; Charles River UK) by ligating the left coronary artery under isoflurane anesthesia and mechanical ventilation (Harvard Apparatus, UK) [16], [17], [18], [29]. Shortly or 4 weeks after generating a MI (successful myocardial infarction was confirmed based on change in ventricular color and cardiac movement), rats received epicardial placement of 100 μl PM-MSC complex. As optimized in the above experiment, PM-MSCs was produced by mixing (i) 40 μl of 2.5% PM resolved in 20 μl 50% sucrose and (ii) 4 × 10^6^ MSCs from derived from fetal membranes of Lewis rats suspended in 40 μl of 1xHBSS (+), by pipetting. A final concentration of 1.0% PM and 10% sucrose was amalgamated. MSCs were labelled with CM-DiI (Molecular Probes, UK) as previously described [16], [17], [18], [29]. The PM-MSC complex was placed onto the epicardial surface of the infarcted heart to cover the infarct and border areas. As a sham control, sham treatment (no cell transplantation) was performed. After each treatment, the chest and skin were closed with suture. Animals were allowed to fully awake under a heat source and were returned to their cages.

### 2.8 Assessment of cardiac function

Transthoracic echocardiography was performed pre-myocardial infarction (base-line) and at Day 14 and 28 post-treatment by the Vevo-770 echocardiography machine (VisualSonics, Netherlands) under isoflurane inhalation administered *via* a nose cone [16], [17], [18], [29]. LV end-diastolic (LVDd) and end-systolic (LVDs) dimensions, under stable, consistent heart rate, were measured using M-mode. Left ventricular ejection fraction (LVEF) was calculated from the data obtained with 2-dimensional tracing. All data were collected blind from at least 3–5 different measurements.

Hemodynamic parameters were measured using cardiac catheterization (SPR-320 and PVAN3.2; Millar Instruments, USA) [16], [17], [18], [29]. Briefly, under general anesthesia using isoflurane inhalation with a nose cone, the catheter was inserted into the left ventricular cavity through the right common carotid artery. Intra-LV pressure signals were measured with a transducer and conditioner (MPVS-300; Millar Instruments) and digitally recorded with a data acquisition system (PowerLab 8/30; ADInstruments, UK). All data were collected from at least 5 different measurements in a blinded manner.

### 2.9 Histological studies

The hearts were harvested, fixed with 4% paraformaldehyde, and frozen in OCT compound using liquid nitrogen. Cryosections were cut and incubated with polyclonal anti–cardiac troponin-T antibody (1:200 dilution; HyTest, Turku, Finland), biotin-conjugated Griffonia simplicifolia lectin I-isolectin B4 (1:100; Vector Laboratories, UK), monoclonal anti-ICAM1 antibody (1:50; AbD Serotec), and monoclonal anti-CD31 antibody (1:50; AbD Serotec), followed by visualization using fluorophore-conjugated secondary antibodies (Life Technologies). Samples were then analyzed by fluorescence microscopy (BZ8000; Keyence) with or without nuclear counterstaining using DAPI (4′, 6-diamidino-2-phenylindole). For semi-quantitative assessments, eight different fields of the border areas (=peri-infarct areas directly surrounding the infarct and containing no obvious cardiomyocyte loss) per heart were randomly selected and assessed. To semi-quantify interstitial collagen deposition, 0.1% picrosirius red (Sigma-Aldrich) staining was performed in another set of sections, followed by assessment by ImageJ analysis software (National Institutes of Health) [16], [18], [29]. In addition, to detect adipogenic and osteogenic differentiation, staining with Oil Red O (Sigma-Aldrich) and Alizarin red (Sigma-Aldrich) was performed, as described previously [17], [18]. Samples were observed and analyzed by light microscopy (BZ8000; Keyence).

### 2.10 Quantitative assessment of donor cell presence in the heart

To quantitatively evaluate the presence of applied male donor cells in the female host heart, the Y chromosome specific *Sry* gene was assessed by real-time PCR (Prism 7900HT; Applied Biosystems, UK) [16], [17]. At 3 and 28 days after implantation of MSCs, the ventricular walls were collected. Genomic DNA was extracted using the DNeasy Blood&Tissue kit (Qiagen), and *Sry* analysis was performed in technical duplicate. The signal in each sample was normalized to the amount of DNA by measuring the autosomal single-copy gene *osteopontin* as an internal standard [16], [17]. To generate a standard curve, the ventricular walls from female rats at 56 days after left coronary artery ligation were mixed with known numbers (1 × 10^7^, 1 × 10^6^, 1 × 10^5^, 1 × 10^4^) of male MSCs (*n* = 3).

### 2.11 Gene expression in the myocardial tissue

Total RNA was extracted from collected cells or from the explanted ventricular walls tissues of rats using the RNeasy Mini Kit (Qiagen) and assessed for myocardial gene expression relevant to the MSC-mediated myocardial repair and protection by quantitative reverse transcription polymerase chain reaction (RT-PCR) (Prism 7900HT; Applied Biosystems, UK) as previously described [17], [18]. TaqMan primers and probes for rat *Hif1a, Il10, Timp1, Mmp2, Igf1, Cxcl12*, and *Fgf2* were purchased from Applied Biosystems. Expression was normalized to *ubiquitin C*. Relative expression to that of the Sham group is presented in the figures.

### 2.12 Statistical analysis

All values are expressed as mean ± SEM. Statistical comparison of two groups was performed using the student's unpaired *t*-test. Other data were statistically analyzed with one-way or two-way ANOVA followed by the Least Significant Difference test to compare groups. A value of *p* < 0.05 was considered statistically significant.

## 3 Results

### 3.1 Characterization of different types of MSCs

To comprehensively demonstrate the feasibility and efficacy of the PM-MSC therapy (epicardial coating of the heart with an instantly-produced PM-MSC complex) *in-vitro* and *in-vivo*, three different types of MSCs were used including rat bone marrow-derived MSCs (Rb-MSCs), human amnion-derived MSCs (Ha-MSCs), and rat fetal membrane-derived MSCs (Rf-MSCs). Rf-MSCs were tested as a model of Ha-MSCs in human cases (isolation of amnion-derived MSCs is technically difficult in rats). Consistent to previous results [8], [27], [29], all of these MSCs showed typical surface marker expression as MSCs, being negative for CD45 and CD34, and positive for CD90, CD29 and CD105 (S.1, S.2, S.3). In addition, all types of MSCs studied were adhered to plastic culture plates and showed a differentiation ability to osteogenic and adipogenic lineages (S.1, S.2, S.3) in response to respective chemical stimulation, fulfilling the criteria to be classified as MSCs [30].

### 3.2 Optimal protocol for instant production of the PM-MSC complex

**Fig. 1:**
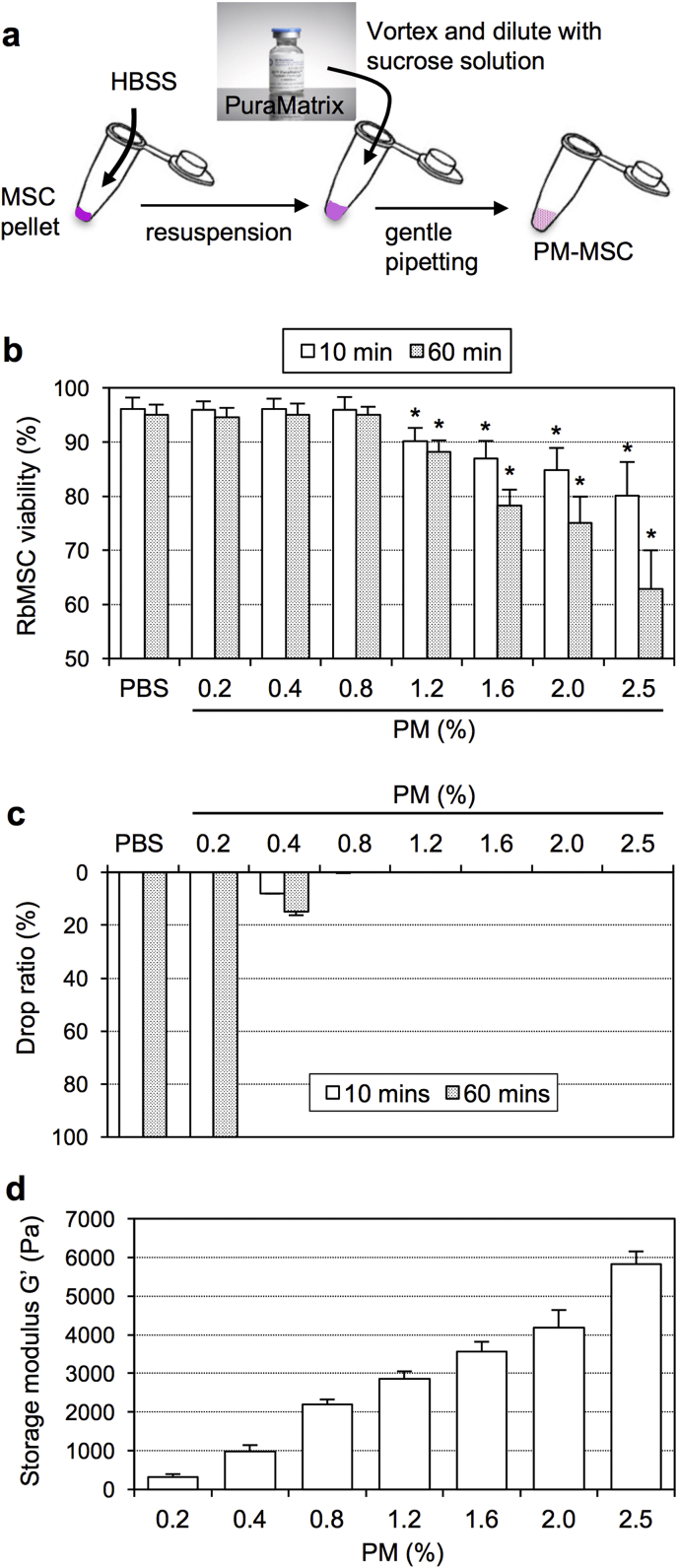
**Optimization of protocol to produce the PM-MSC complex**. **(a)**. A scheme for production of the PM-MSC complex is shown. MSCs were suspended in HBSS and then gently mixed by pipetting with PM that was diluted with sucrose solution following sonication. **(b)**. Viability of rat bone marrow-derived MSCs at 10 and 60 min after mixture with different doses of PM *in vitro* was assessed by trypan blue staining. Over 1.2% PM showed toxicity to MSCs. n=4 in each point; *p<0.05 vs. corresponding value of the PBS group. **(c)**. Retention of the PM-MSC complex (rat bone marrow-derived MSCs in different doses of PM) was assessed by a tilting test on the plastic plate *in-vitro*. Over 0.8% PM allowed appropriately retention. n = 4 i*n each point*. **(d)**. Storage modulus G′ was measured in the PM-MSC complex with different PM doses (at 10 min after mixture). *n=4 in each group*. (For interpretation of the references to colour in this figure legend, the reader is referred to the web version of this article.)

First, we explored the practical procedure and optimal dose of PM to produce the most effective PM-MSC complex for coating the heart surface. It is presumed that an excessively high dose of PM may be toxic to MSCs, while a dose that is too low will mean that the PM is too soft to correctively adhere to the surface of the heart. Thus, we compared MSC viability and retention of the PM-MSC complex among ranging doses of PM (0.2–2.5%) in *in-vitro* models. For the donor cell, rat bone marrow-derived MSCs were used in the first instance. To mix the PM solution and MSCs, we identified it to be practical to combine 40 μl PM solution (diluted with sucrose solution [final concentration of 10%] following sonication to reduce its viscosity) with 40 μl MSC suspension (1 × 10^6^ cells in HBSS) (Fig. 1a). Sucrose was used to enhance cell viability according to the company's instruction (3D Matrix, Inc), and HBSS buffer was used to provide the isotonic, neutral conditions for MSCs. The final PM-MSC volume of 80 μl was chosen as the most suitable volume to cover the heart of a 250 g rat. PM solution did not undergo gelation by mixture with sucrose solution, but did initiate gradual gelation after the mixture with MSC/HBSS suspension. We found a higher PM dose offered a greater gel strength, which may be advantageous to retain MSCs inside the hydrogel. For all PM doses studied (0.2–2.5%), the PM-MSC complex was soft enough for at least 20 min after mixing to still be easily handled with a pipette, which ensures that the PM-MSC complex remains easy to handle clinically at the time of placement. The viability of MSCs was maintained for a minimum of 1 h when the final PM dose was lower than 1.2%, compared to MSCs cultured in PBS (Fig. 1b). In contrast, PM doses higher or equal to 1.2% reduced the MSC viability by 10 min after mixing, which was further decreased by 1 h. Conversely, when the PM dose was less than 0.4%, the resulting PM-MSC complex could not retain its form on a tilted plate, suggesting an impaired capability for epicardial coating (Fig. 1c). As a result of rheological assessment, storage modulus G′ of the PM-MSC complex was found to increase proportionally to its PM dose. The storage modulus G′ of 0.8% PM-MSC was approximately 2000 Pa (Fig. 1d).

We next investigated whether the above findings are applicable to human amnion-derived MSCs, which we aim to use in our future clinical trials. The results agreed with the conclusion using rat bone marrow-derived MSCs as above (Fig. 1); 0.8–1.0% PM did not reduce human amnion-derived MSC viability, but allowing sufficient retention of a PM-MSC complex on the tilted plate (S. 4). Additional tests using rat fetal membrane-derived MSCs also demonstrated the same results of MSC viability and retention of the PM-MSC complex (S. 4). In both cell types, handling of the 0.8–1.0% PM-MSC complex was easy with usual pipetting. Collectively, we selected 0.8–1.0% of PM to be the optimal dose to achieve epicardial coating of the heart, which was used for further experiments in this study.

### 3.3 Acidity of the PM-MSC complex

**Fig. 2:**
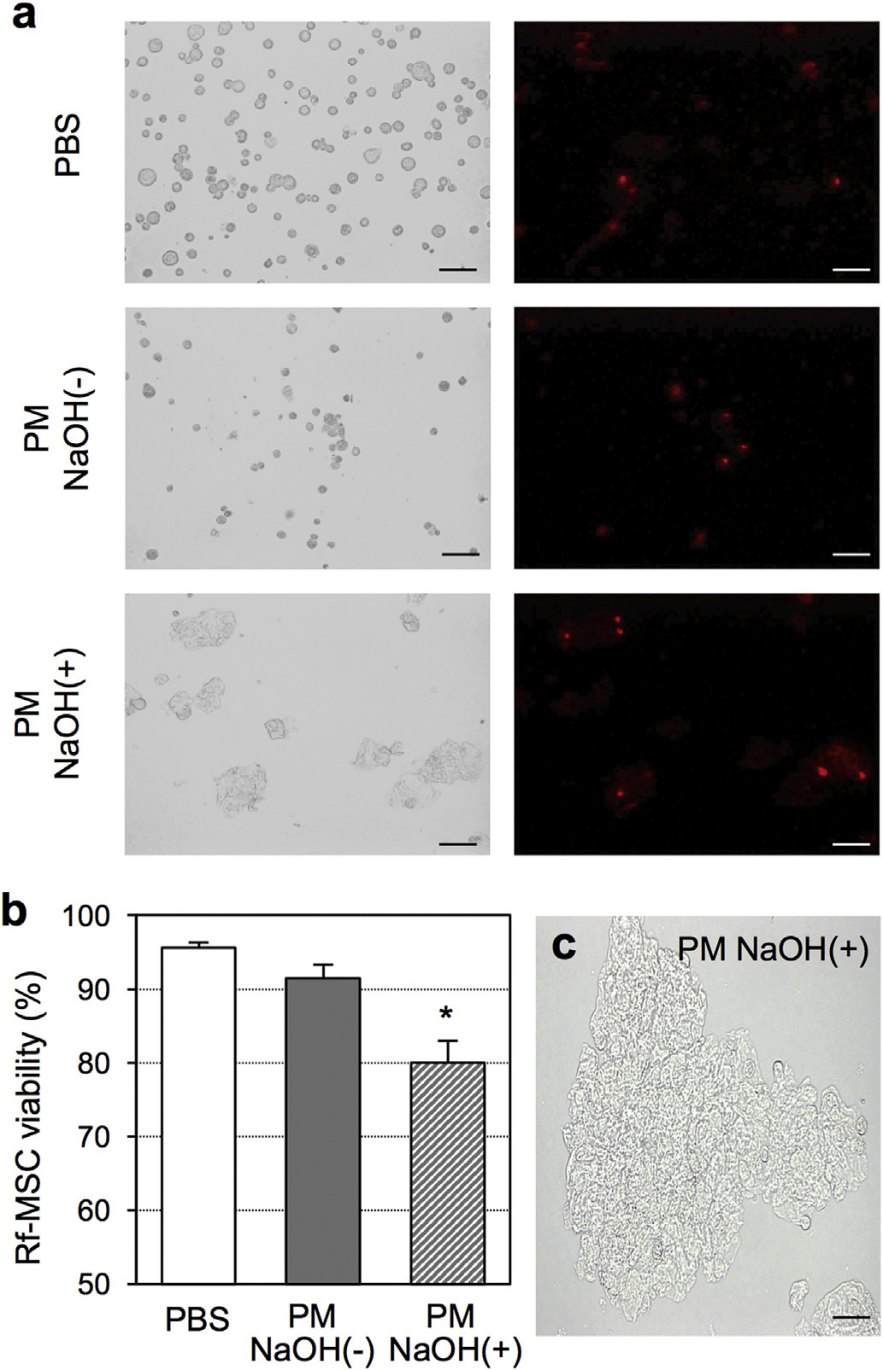
**Neutralization of the PM-MSC complex**. **(a, b)** Viability of rat fetal membrane-derived MSCs at 10 min after mixture of PM (final dose 0.8%) with or without neutralization using NaOH *in-vitro* was assessed by propidium iodide staining. Representative pictures in each group are presented (a). Scale bar = 50 μm. The bar graph presents the averaged data (b). *n=4 in each point*; *p<0.05 vs. the PBS group. **(c)**. Representative phase-contrast microimage of heterogeneous “compacted” gelation caused by neutralization (PM NaOH(+)). Scale bar = 50 μm.

The PM solution is known to be acidic before gelation. During optimization of the PM-MSC complex, we became aware that the gelated complex remained acidic (pH 4.0–5.0 when the PM dose was between 0.5 and 1.0%). Although the viability of incorporated MSCs was not reduced in such acidic complex for 1 h after mixture (Fig. 1a, Fig. S4), neutralization may further improve viability and functionality of MSCs within the PM-MSC complex. We therefore tried to neutralize the PM-MSC complex by the addition of NaOH solution to the PM solution prior to the addition of rat fetal membrane-derived MSC solution. With this method, we were able to achieve a pH of 7 in both the PM solution and the resulting PM-MSC complex. However, contradictory to our expectation, this neutralization exacerbated the death of MSCs in the PM-MSC complex as measured by using propidium iodide staining (Fig. 2a and b) as well as by the Live and Dead Cell assay (S. 5). It was speculated that the addition of NaOH altered the process of gelation of PM, resulting in “compacted” aggregation formation of the hydrogel (Fig. 2c) and limiting the survival of MSCs incorporated in it.

**Fig. 3:**
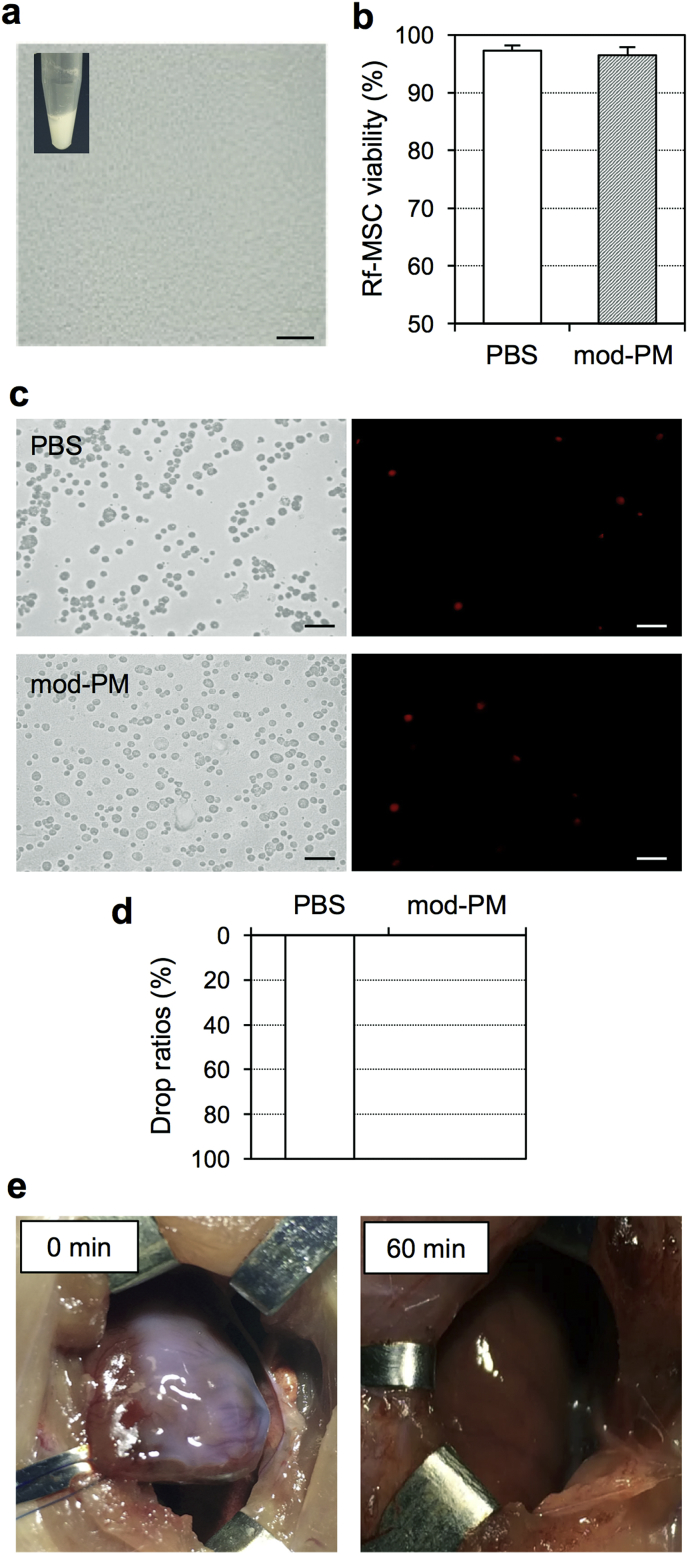
**Production and characterization of modified PM**. **(a)**. Appearance and microimage of produced homogenous, neutral PM are shown. Scale bar = 50 μm. **(b, c)**. Viability of rat fetal membrane-derived MSCs at 10 min after mixture with modified PM (mod-PM; final dose 0.8%) *in-vitro* was assessed by propidium iodide staining (b). *n=4 in each point.* Representative pictures of propidium iodide in each group are presented in (c). Scale bar = 50 μm. **(d)**. Retention of the modified PM-MSC complex was assessed by a tilting test on the plastic plate *in-vitro. n=4 in each point.***(e)**. Retention of the modified PM-MSC complex on the beating rat heart *in-vivo.* Retained modified PM-MSC complex (white) at 0 min dissociated by 60 min after epicardial placement. Representative pictures from n = 4 are presented.

We then tried to break up the compacted PM hydrogel by neutralization. The heterogeneously compacted PM hydrogel, which was produced by a mixture of the PM solution and NaOH, was broken down using sonication, resulting in formation of smaller and homogenous PM particles (Fig. 3a) at a pH of 7.0 ± 0.0 (n = 5). In contrast to the ordinary PM hydrogel that is transparent, this modified PM was white-milky opaque appearance. We next mixed a suspension of rat fetal membrane-derived MSCs with this homogenous modified-PM product, using a final PM dose of 1.0%. The pH of the modified-PM-MSC complex was 7.0 ± 0.1 (n = 5). Propidium iodide staining (Fig. 3b and c) and Live and Dead Cell assay (S. 6) consistently demonstrated that the viability of MSCs in modified-PM-MSC complex was sufficiently high, being equivalent to that cultured in PBS. In addition, the mixture was also successful in retaining its form on a tilted plate *in-vitro* (Fig. 3d). However, it was revealed that this altered complex could not sufficiently retain on the beating rat heart *in-vivo*. The modified PM-MSC complex was easily handled, spread and placed on the heart surface by simple pipetting, but by 60 min (with the chest temporarily closed), this complex had almost fully disappeared from the heart surface. This was thought to be either due to dilution by surronding fluid or by mechanical displacement by the surrounding tissues (Fig. 3e).

### 3.4 Retention of MSCs on the heart surface after epicardial coating with instant PM-MSC

Although our challenge to neutralize the pH of the PM-MSC complex was not successful, safety of the original (acidic) PM has been proven in aninmals and human patients [26], [31]. It is thought that PM is neutralized by surrounding physical fluid soon after *in-vivo* implantation [32]. We therefore decided to investigate the efficacy of the acidic PM-MSC complex to achieve retention and survival of MSCs on the heart surface *in-vivo*.

**Fig. 4:**
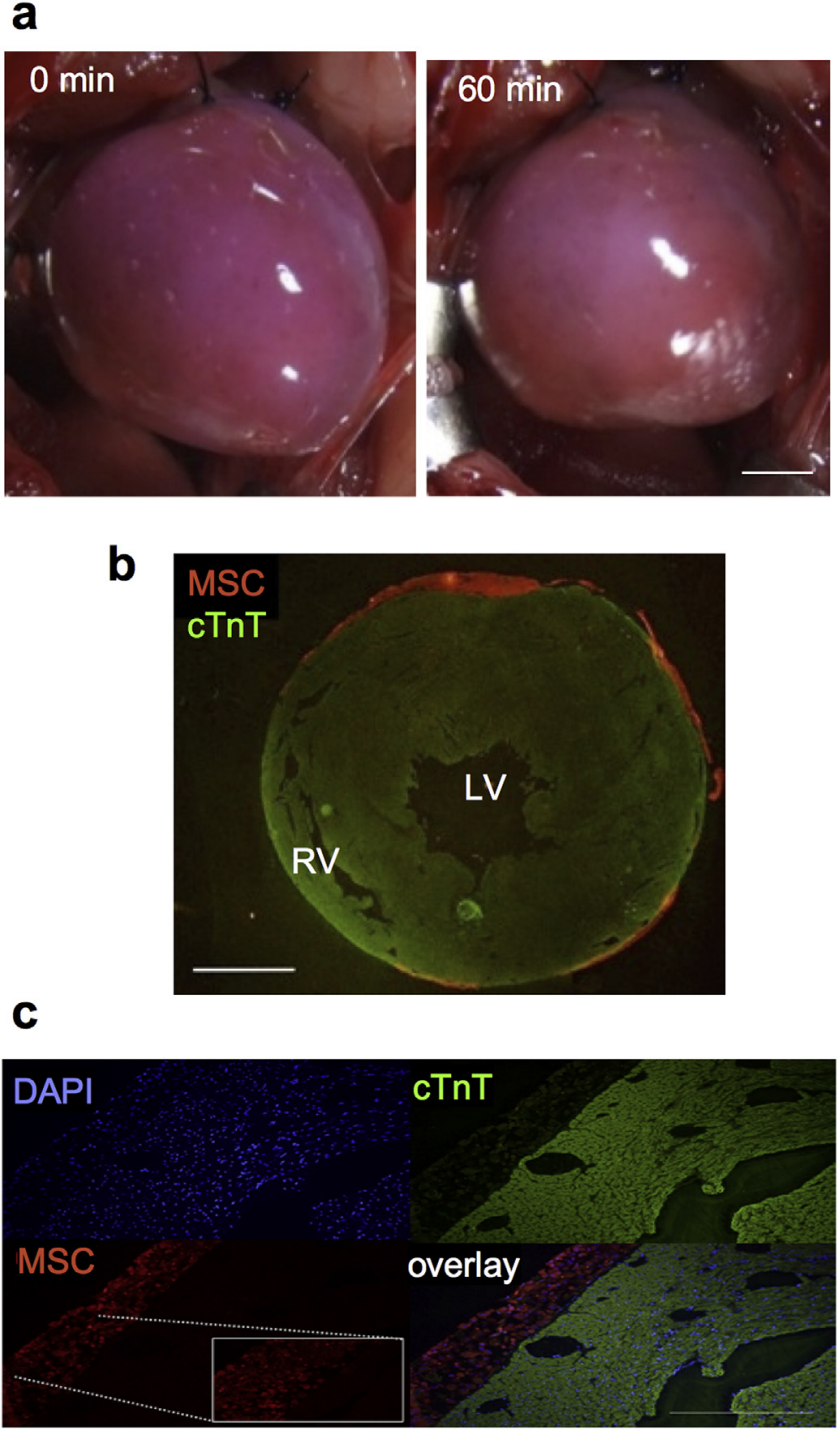
**In-vivo retention of the PM-MSC complex on the rat beating heart**. **(a)**. Retention of the PM-MSC complex (1 × 10^6^ rat bone marrow-derived MSCs in 0.8% PM) on the beating rat heart *in-vivo* was assessed at 0 and 60 min after drop-by-drop spread. MSCs were labelled with a red fluorescent dye, CM-DiI, visualizing the complex in pink. Representative pictures from n = 4 are presented. *Scale bar=5 mm*. **(b, c)**. One day after epicardial placement, immunofluorescence demonstrated that the PM-MSC complex (orange) tightly attached to the heart surface, suitably covering the LV walls. *Scale bar=5 mm; LV, left ventricle; RV; right ventricle; cTnT; cardiac Troponin T; DAPI; 4′,6-diamidino-2-phenylindole*. (For interpretation of the references to colour in this figure legend, the reader is referred to the web version of this article.)

The above optimized PM-MSC complex (0.8% PM including 1 × 10^6^ MSCs with a total volume of 80 μl) was instantly produced and directly spread onto the epicardial surface of the beating heart of a syngeneic rat by a drop-by-drop pipetting under left thoracotomy and pericardiotomy. All of these procedures were technically straightforward and resulted in a successful, homogenous coating of the epicardial heart surface with the PM-MSC complex (Fig. 4a). Of note, the PM-MSC complex adhered to the heart surface and was retained firmly without dissociation for 60 min after transplantation (with the chest closed). Furthermore, histological observation confirmed the successful retention of MSCs at one day after epicardial coating with instant PM-MSC complex (Fig. 4b and c).

### 3.5 Therapeutic effects of epicardial coating with the instant PM-MSC complex

**Fig. 5:**
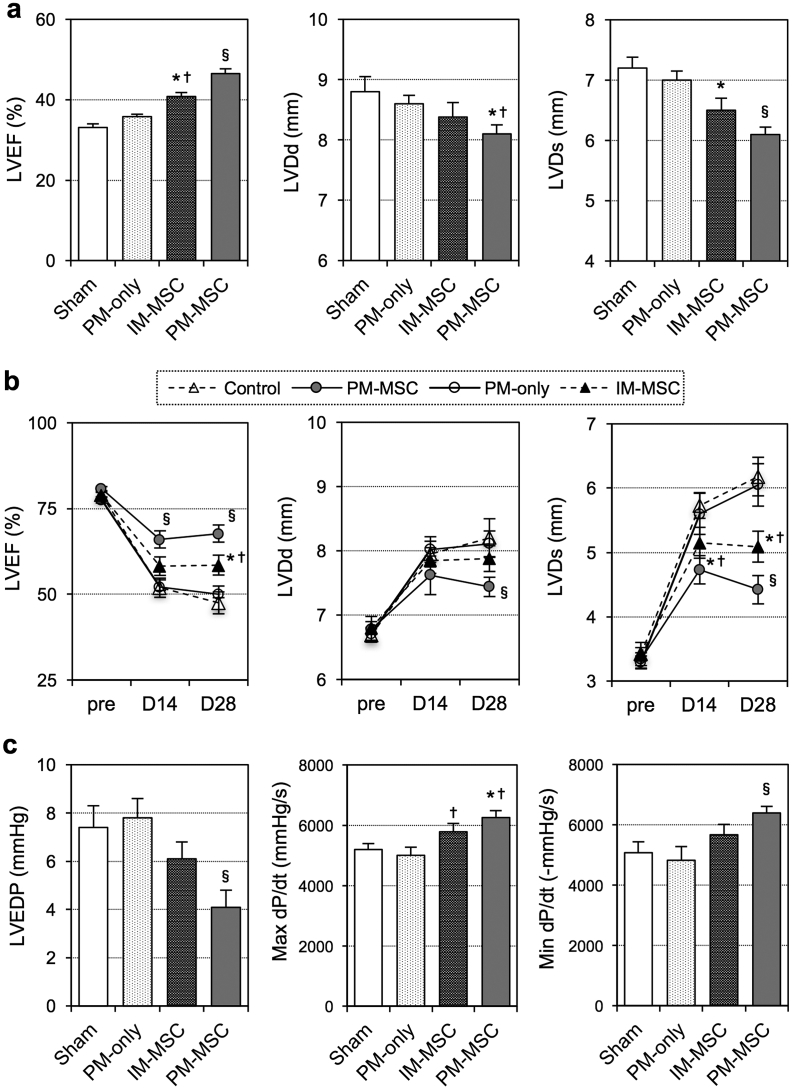
**Cardiac function improvement by epicardial PM-MSC coating in post-MI ischemic heart failure and acute MI models**. **(a)**. At 4 weeks after coronary artery ligation, rats received epicardial coating with the instant PM-MSC complex (PM-MSC group; 1 × 10^6^ rat bone marrow-derived MSCs in 0.8% PM) or intramyocardial injection of suspension of 1 × 10^6^ rat bone marrow-derived MSCs (IM-MSC group). Sham (open chest only at 4 weeks after coronary artery ligation) and epicardial placement of 0.8% PM mixed with HBSS (no MSC; PM-only group) were added as controls. At 4 weeks after treatment, echocardiography demonstrated reduced cardiac dimension and improved cardiac function in the PM-MSC group. LVEF, LV ejection fraction; LVDd/s, LV dimension at end-diastole/end-systole; n=9∼11; *p<0.05 vs. the Sham group,^*✝*^*p<0.05 vs. PM-only group,*^*§*^*p<0.05 vs. all Sham, PM-only and IM-MSC groups.***(b, c)**. Immediately after coronary artery ligation, rats received epicardial coating with the instant PM-MSC complex (PM-MSC group; 1 × 10^6^ rat fetal membrane-derived MSCs in 0.8% PM) or intramyocardial injection of suspension of 1 × 10^6^ rat bone marrow-derived MSCs (IM-MSC group). Sham (MI only) and epicardial placement of 0.8% PM mixed with HBSS (no MSC; PM-only group) were also studied. At Day 14 and 28 post-treatment, echocardiography demonstrated reduced cardiac dimension and improved cardiac function in the PM-MSC group (**b**). At Day 28 post-treatment, catheterization showed improved cardiac function in the PM-MSC group (**c**). LVEDP, LV end-diastolic pressure; n=8 in each group; *p<0.05 vs. the Sham group,^*✝*^*p<0.05 vs. PM-only group,*^*§*^*p<0.05 vs. all Sham, PM-only and IM-MSC groups.*

Subsequently, we investigated the therapeutic effect of epicardial coating with the instantly-produced PM-MSC complex in a rat model of post-MI ischemic cardiomyopathy. At Day 28 after left coronary artery ligation, the optimized PM-MSC complex using rat bone marrow-derived MSCs was produced (Section 3.2 (Fig. 1a)) and spread onto the rat epicardial heart surface using the protocol outlined above (Section 3.4). It was visually confirmed that the placed PM-MSC complex promptly and effectively adhered to the beating heart surface using MSCs labelled with red fluorescent dye (CM-DiI). Techniques for production and spread of the PM-MSC complex were confirmed to be straightforward and reproducible. There were no complications, including arrhythmogenesis, associated with the PM-MSC coating. At Day 28 post-treatment, echocardiography demonstrated that epicardial coating with the instant PM-MSC complex enhanced global cardiac function and decreased ventricular dilatation, compared to intramyocardial MSC injection and sham control (Fig. 5a; n = 9–11 in each group). Epicardial coating with PM only (without MSCs) did not affect cardiac function or dimension.

Therapeutic efficacy of instant epicardial PM-MSC coating was additionally confirmed using a second rat model, in which rat fetal membrane-derived MSCs were transplanted to a syngeneic rat with acute MI. Shortly after left coronary artery ligation under pericardiotomy, the PM-MSC complex was transplanted onto the rat epicardial heart surface using the same optimized protocol. Again, placed PM-MSC complex promptly and stably adhered to the beating heart, with no complications. At Day 14 and/or 28 after the PM-MSC therapy, echocardiography revealed improved cardiac function with preserved left ventricular dimensions compared to all other groups (Fig. 5b; n = 8 in each group). We found a consistent and significant improvement in cardiac function and hemodynamic status by cardiac catheterization at Day 28 post-epicardial PM-MSC treatment (Fig. 5c; n = 8). The lower LV end-diastolic pressures in the PM-MSC-treated hearts may suggest that epicardial coating of the heart with the PM-MSC complex did not result in a constrictive physiology. Intramyocardial injection of MSCs showed improvement of cardiac function compared to the sham group, but the degree of the improvement was not as great as the PM-MSC group. Epicardial placement of PM only did not change cardiac function.

### 3.6 Factors underlying improved effect of MSC-therapy by epicardial PM-MSC coating

**Fig. 6:**
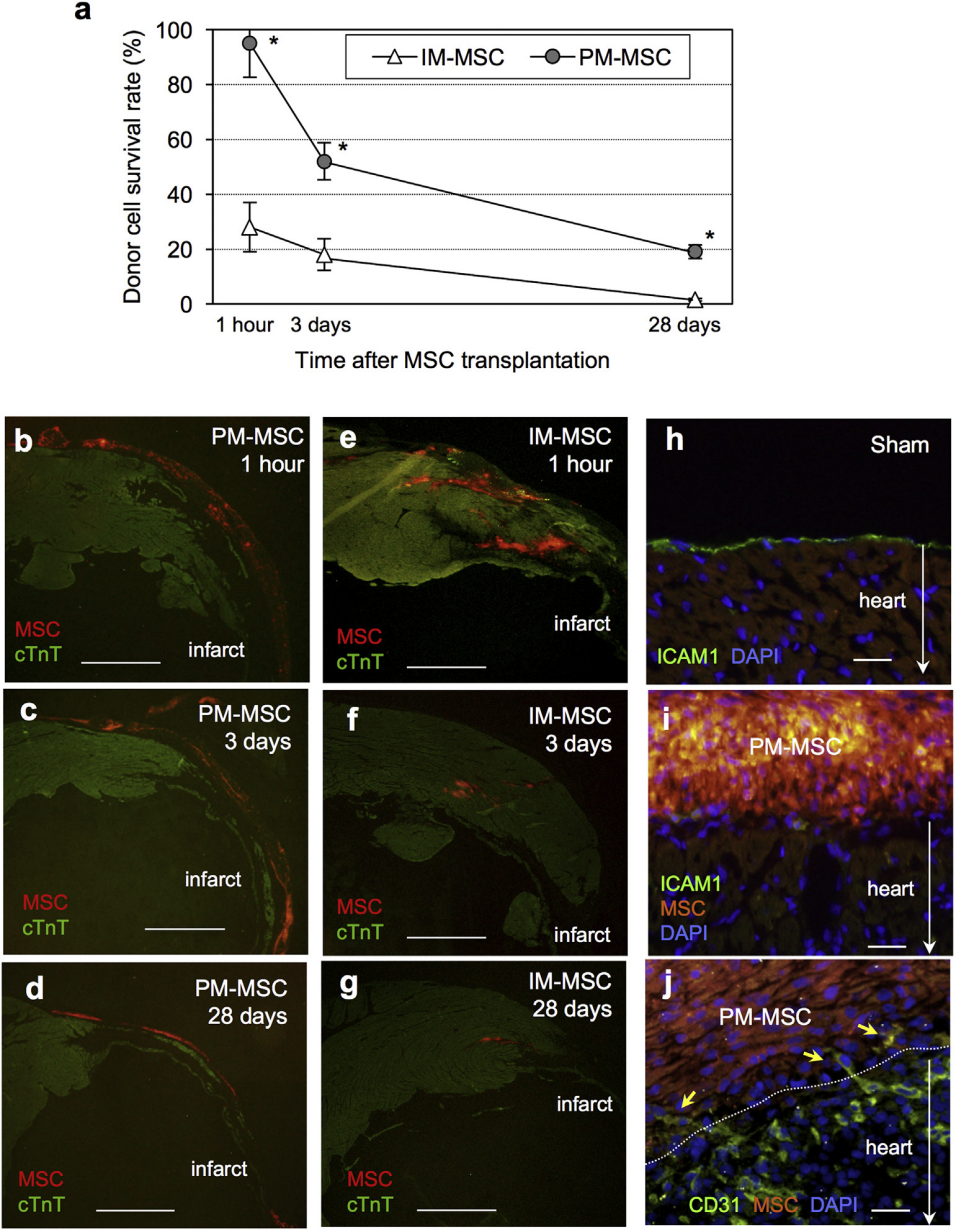
**Improved donor cell survival by epicardial PM-MSC coating**. **(a)**. At 4 weeks after coronary artery ligation, rats received epicardial PM-MSC coating (PM-MSC group; 1 × 10^6^ male rat bone marrow-derived MSCs in 0.8% PM) or intramyocardial injection of suspension of 1 × 10^6^ male rat bone marrow-derived MSC (IM-MSC group). Quantitative PCR for the male-specific *sry* gene showed that the PM-MSC group markedly enhanced initial retention and survival of donor cells, compared to the IM-MSC group. n=4∼5 in each point; *p<0.05 vs. the IM-MSC group.**(b**–**d)**. Immunofluorescence demonstrated that the PM-MSC complex (orange; labelled with CM-DiI) covered the surface of LV walls (both peri-infarct and infarct areas) at 1 h (b), Day 3 (c), and Day 28 (d). The majority of surviving MSCs retained on the heart surface. Scale bar = 1 mm **(e**–**g)**. Immunofluorescence demonstrated that donor cells in the IM-MSC group were found forming isolated cell-clusters (orange; labelled with CM-DiI) at 1 h (e), Day 3 (f), and Day 28 (g). The surviving donor cell numbers in the IM-MSC group appeared to be smaller compared to the PM-MSC group. Scale bar = 1 mm **(h**–**j)**. Immunolabeling detected a monolayer of epicardial cells (green) on the surface of heart in the Sham group (h). The epicardial cells disappeared by one day after PM-MSC placement (i). There were sprouting vessels (green) from the heart into the PM-MSC complex by Day 3 after placement (j). Scale bar = 50 μm. (For interpretation of the references to colour in this figure legend, the reader is referred to the web version of this article.)

The potential underlying mechanisms for the enhanced therapeutic effects of epicardial PM-MSC coating in the treatment of post-MI ischemic cardiomyopathy were investigated using Rb-MSCs. Of note, the qPCR-based quantitative assessment of donor cell presence showed that retention and survival of MSCs were markedly enhanced by epicardial PM-MSC coating (Fig. 6a). The initial retention of MSCs was almost 100% (to the total transplanted MSC number) at 1 h post-epicardial PM-MSC coating, while it was approximately 35% in the case of intramyocardial injection of MSC suspension. Subsequent donor cell survival rates at Day 3 and 28 were markedly increased by epicardial PM-MSC coating. Histology confirmed this observation; the donor cell presence after epicardial PM-MSC coating appeared to be greater, compared to intramyocardial injection, in every time point studied (Fig. 6b–g). The majority of donor MSCs persisted on the heart surface without migration into the host cardiac tissues up to Day 28 after epicardial PM-MSC coating, while intramyocardially injected MSCs formed isolated cell clusters within the myocardium. Notably, the epicardial cell barrier, which was found in the heart of the Sham group (Fig. 6h), disappeared by Day 1 after PM-MSC coating (Fig. 6i) with formation of connecting vasculature between the PM-MSC complex and host myocardium (Fig. 6j). This feature may suggest communication between donor MSCs and the host myocardium, allowing the paracrine signaling from MSCs to effect myocardial repair.

**Fig. 7:**
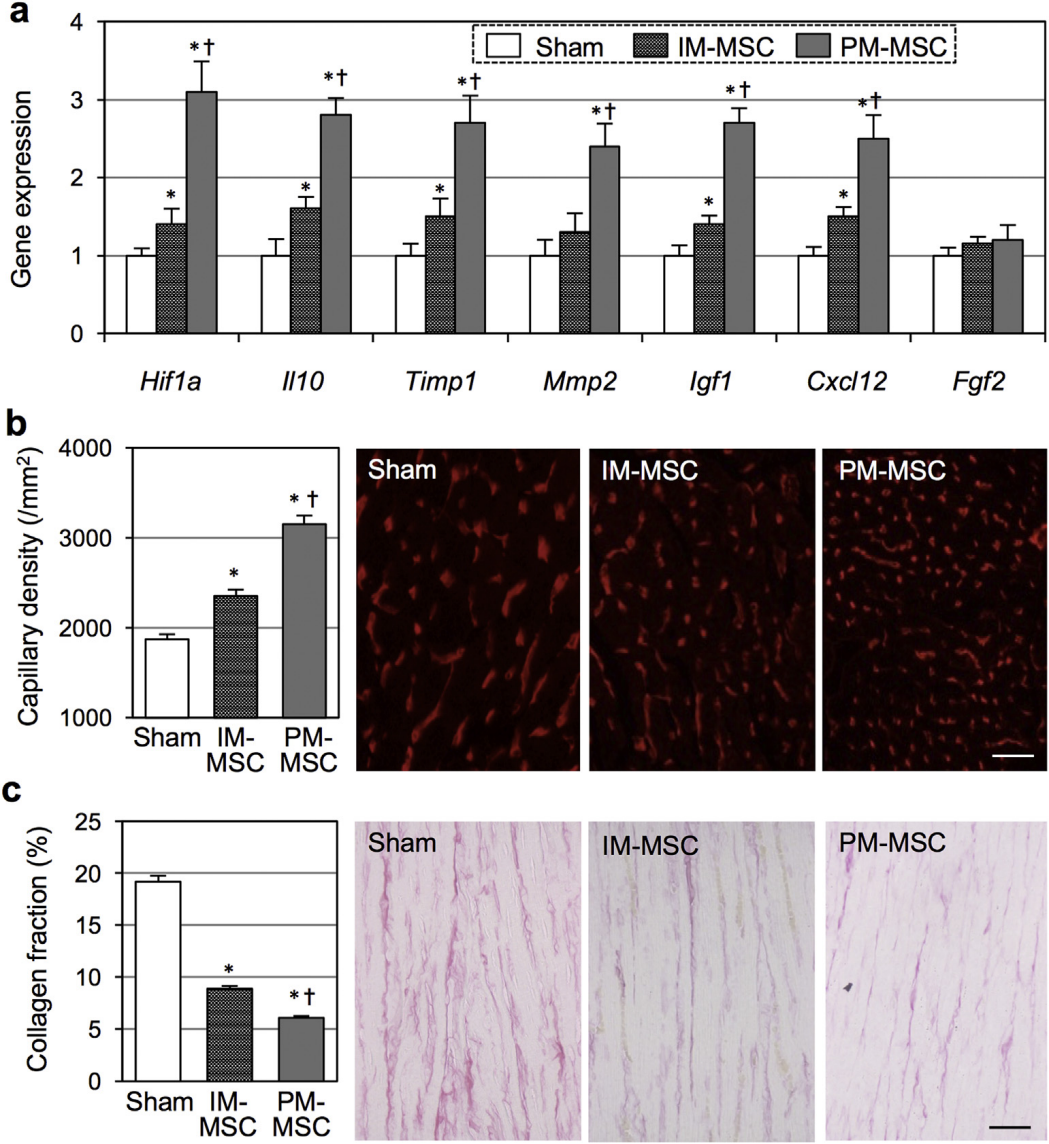
**Underlying factors of effects of epicardial PM-MSC coating for heart failure**. At 4 weeks after coronary artery ligation, rats received epicardial PM-MSC coating (PM-MSC group; 1 × 10^6^ male rat bone marrow-derived MSCs in 0.8% PM), intramyocardial injection of suspension of 1 × 10^6^ male rat bone marrow-derived MSC (IM-MSC group) or Sham treatment. In (a)–(c), *p<0.05 vs. the Sham group,^*✝*^*p<0.05 vs. the IM-MSC group. Scale bar=50 μm (b) and 100 μm (c).***(a)**. Quantitative RT-PCR showed that the PM-MSC group upregulated a group of genes that are relevant to myocardial repair compared to the Sham and IM-MSC groups at Day 3 after treatment. Expression in the Sham group was arbitrarily defined as 1.0. *n=4 (Sham group), 5 (IM-MSC group) and 5 (PM-MSC group)*. **(b)**. Isolectin B4 staining revealed increased capillary density in the peri-infarct viable myocardium (=myocardial areas directly surrounding the infarct and containing no obvious cardiomyocyte loss) at Day 28 in the PM-MSC group compared to the IM-MSC and Sham groups. *n=4 in each group*. **(c)**. Picrosirius red staining showed reduced interstitial collagen deposition in the peri-infarct viable myocardium at Day 28 in the PM-MSC group compared to other groups. n = 4 in each group. (For interpretation of the references to colour in this figure legend, the reader is referred to the web version of this article.)

Consistent to this observation of enhanced donor cell survival, additional analyses revealed that epicardial PM-MSC coating augmented the paracrine effect of the MSC-based therapy. Real time RT-PCR demonstrated enhanced upregulation of a range of anti-inflammatory and tissue repair-related genes, including *Hif1a, Il10, Timp1, Mmp2, Igf1* and *Cxcl12*, in the myocardium following epicardial coating with the PM-MSC complex, compared to intramyocardial MSC injection and the Sham group (Fig. 7a). Histology demonstrated an increase in microvascular formation and a reduction of interstitial collagen deposition in the peri-infarct area being achieved by intramyocardial MSC injection compared to the sham group. These benefits were further amplified by epicardial coating with the PM-MSC complex (Fig. 7b and c).

## 4 Discussion

This study demonstrated that a new approach for MSC-based therapy, namely the epicardial “coating” of the heart with a self-assembling peptide hydrogel incorporating MSCs, has the potential to refine MSC-based therapy towards clinical success. By comparing cell toxicity, ease of handling, and retention of MSCs, we first optimized the protocol to generate the PM-MSC complex (final dose of 0.8–1.0% of PM) using different types of rat and human MSCs. The PM-MSC complex with lower than 0.8% PM could not retain on the beating heart, while higher than 1.0% PM showed toxicity to donor MSCs. The optimized PM-MSC complex was soft enough to handle with a pipette for at least 20 min after the mixture of the PM solution and MSC suspension. Upon spreading of the mixture onto the beating heart, this PM-MSC complex promptly and homogenously adhered without being displaced. The PM-MSC coating therapy enhanced cardiac function and structure in rat models of both acute MI and post-MI ischemic cardiomyopathy. Importantly, the degree of these therapeutic effects was superior to the current standard method of intramyocardial injection of MSC suspension. These amplified effects correlated with increased donor cell retention and survival, which enabled augmentation of paracrine signaling from MSCs. When compared to intramyocardial MSC injection, epicardial PM-MSC coating reduced fibrosis and increased neovascular formation in the viable but failing peri-infarct areas, and was associated with upregulation of a range of reparative genes. All of the procedures for the epicardial placement of the PM-MSC complex were found to be straightforward even in small rodent models.

Epicardial placement is a relatively new cell-delivery route to the heart [17], [33], [34]. This method can be achieved by the use of cell-sheets or biomaterials, including fibrin sealants or collagen sponge [29], [33], [34], [35]. There is increasing evidence to show that this route has significant advantages over the current injection methods both in terms of greater donor cell retention/survival and reduced complications such as arrhythmogenicity or coronary artery embolism [13], [16], [29]. This current study attempted to upgrade the epicardial placement to epicardial “coating” of the heart with instantly-produced hydrogel-MSC complex. This method has important merits over epicardial placement of cell-sheets or pre-made constructs of stem cell-incorporating biomaterials, in being more accessible to clinicians and hospitals. As the PM-MSC complex is produced instantly on the site and time of the treatment using MSC suspension and PM solution delivered by the hub cell processing hub center and PM-manufacturing company, the hospital does not need to be equipped with a GMP-cell culture facility. There is also no need for the extra labor/cost-consuming steps involved in GMP-production of cell-sheets or pre-made constructs. These features enable this treatment to be practical in any cardiac surgical department, allowing this cell-therapy to become a standard, ubiquitous treatment. In this study, we were able to highlight the great potential of the PM hydrogel to realize this approach. Ease of handling, gelation speed, and hydrogel strength of the PM was suitable to enable epicardial coating with MSCs. The PM product is produced under GMP conditions and has been given a CE mark for hemostasis. As such, the PM-MSC therapy for heart failure is a repurpose of PM, suggesting an economical and straightforward development, including regulatory approval process. Fibrin sealants might be able to achieve similar epicardial coating. However, because the PM is fully synthetic, it obviates issues associated with fibrin sealants, including heterogeneous products and risks associated with human/animal materials [36]. In addition, compared to the PM method that needs to spread only one solution (PM-MSC complex) onto the heart, the fibrin sealant-based method will be more complicated due to requirement to manage two different solutions (thrombin and fibrinogen solutions).

As a realistic strategy of clinical application of epicardial PM-MSC coating therapy, we propose a combination with coronary artery bypass grafting (CABG). At the moment, it is more reliable and straightforward to conduct this innovative cell-therapy to an exposed heart during cardiac surgery. Although CABG is routinely performed to treat post-MI ischemic cardiomyopathy, its benefits are not always convincing [37]. Thus improvement of this treatment would benefit a large number of patients. An addition of cell therapy has been proven to augment the efficacy of CABG [38]. It is speculated that increased myocardial blood flow by CABG and improved microvascular formation by the PM-MSC therapy will achieve more functional improvement of myocardial perfusion. This will in turn improve donor MSC survival and also prevent CABG graft failure, leading to further synergistic improvements of the overall therapeutic efficacy. Epicardial PM-MSC coating can be easily completed by any surgeon without adding stress to patients. Although this MSC-based therapy, even combined with CABG, may not completely normalize the injured heart, it could offer heart failure patients a significant enhancement in survival ad improved quality of life. This will be of great value to patients with heart failure, half of whom die within 2–3 years [39]. By combining with CABG, costs for surgical procedures, pre-/post-operative assessments, and follow-up will be shared and reduced. This user-friendly strategy (epicardial coating of the heart with the instant PM-MSC complex at time of CABG) therefore has potential to become an ordinary, routine treatment for ischemic cardiomyopathy.

The choice of the source of MSCs is another factor determining success of MSC therapy. This study demonstrated that both bone marrow-derived MSCs and fetal membrane-MSCs are suitable for the PM-MSC epicardial coating therapy in rat models. Although bone marrow-derived MSCs have been more extensively investigated in experimental and clinical studies, fetal membrane-derived MSCs (amnion-derived MSCs in the clinical case) have several important advantages. Above all, the amnion can offer a much greater initial yield of MSCs [7] allowing easier large-scale production with reduced risk of deterioration of MSC-quality and functionality due to excessive passaging. This advantage is critical to commercializing an MSC product. Unlike bone marrow, the amnion can be collected from clinical waste with the donor/volunteer subjected to invasive procedures like bone marrow aspiration. Additionally, amnion-derived MSCs have extensive immunosuppressive abilities, enabling even xenogeneic (human to rat) cell transplantation without immunosuppression [40]. Efficacy of fetal membrane-derived MSCs for the treatment of heart failure has been reported to be equivalent to that of bone marrow-derived MSCs [28].

We could not successfully neutralize the PM-MSC complex before implantation. Despite this, the results of *in-vivo* studies clearly demonstrated that epicardial coating with the “acidic” PM-MSC complex offered enhanced donor cell survival and superior therapeutic effects to that by the current standard method (intramyocardial injection of MSC suspension). It was speculated that the implanted PM-MSC complex was naturally neutralized by the surrounding pericardial fluid soon after closing the chest. This has previously been demonstrated in the application of PM for hemostasis [26]. It will be interesting to monitor the change in acidity of the PM-MSC after *in-vivo* epicardial coating in future studies. It may be that successful neutralization of the PM-MSC complex results in further enhancement of donor cell survival and therapeutic effects. An option may be the use of another type of self-assembling peptide hydrogel, which is originally neutral [41]. Further pre-clinical development is required before application of the epicardial PM-MSC coating therapy to patients.

## 5 Conclusion

In conclusion, our *in-vitro* and *in-vivo* studies provide proof-of-concept data indicating that epicardial coating of the heart with the instantly-produced PM-MSC complex is a promising, innovative therapy for the treatment of heart failure. This widely-accessible approach has the potential to develop MSC-therapy into an established generic treatment for heart failure. As safety of the PM hydrogel in the use for hemostasis has been clinically proven, its repurpose here represents a low-risk development. Application of this method for the treatment of other organ diseases may also be promising. Further pre-clinical and clinical development of this advanced cell-therapy approach is encouraged.

## Figures and Tables

**Fig. s1:**
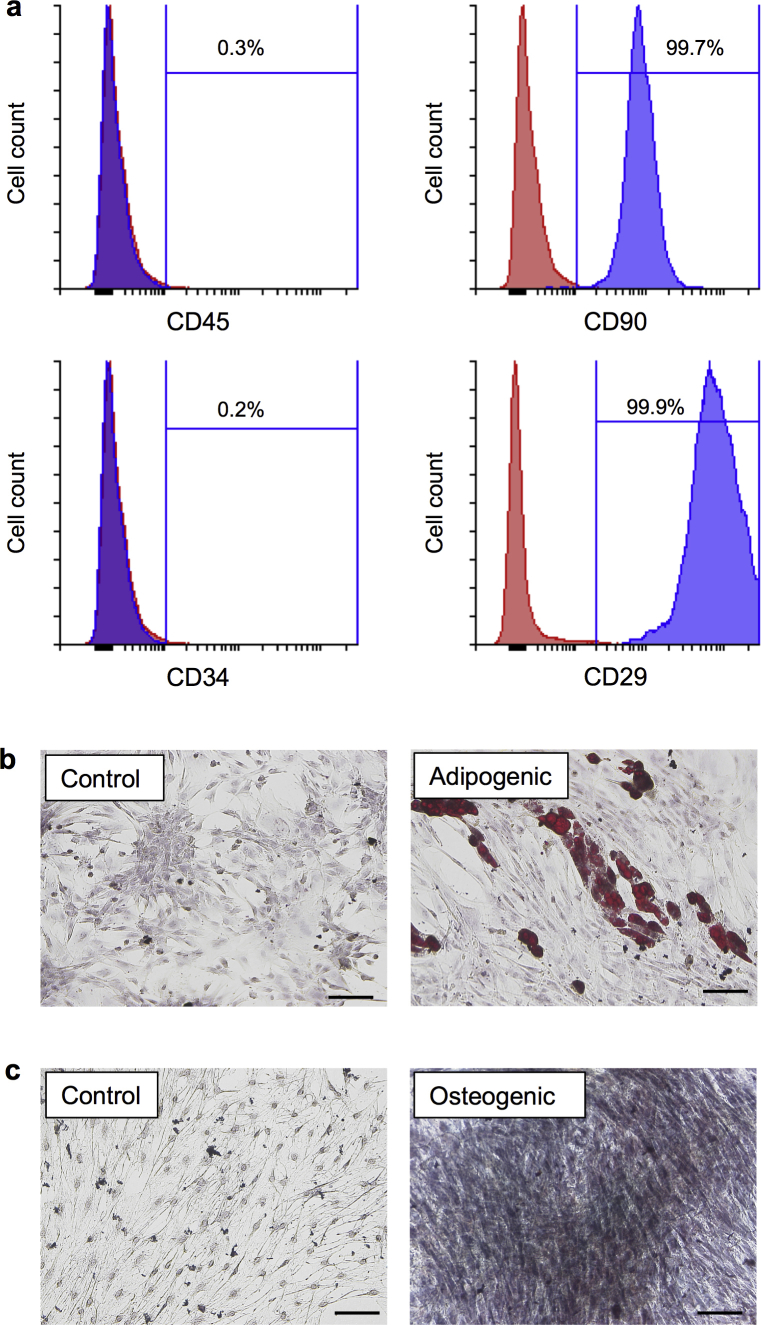
**Characterization of rat bone marrow-derived (Rb) MSCs**. **(a)**. CD marker expression of rat bone marrow-derived MSCs (passage 4) was assessed by flow cytometric analysis. Red; IgG control, Blue; CD45/90/34/29 staining. **(b,c)**. Differentiation of rat bone marrow-derived MSCs to adipocyte (b; Oil Red-O staining) and osteocytes (c; Alizarin red staining) was confirmed. Control; cultured in usual medium; Scale bar = 100 μm (b) and 50 μm (c).

**Fig. s2:**
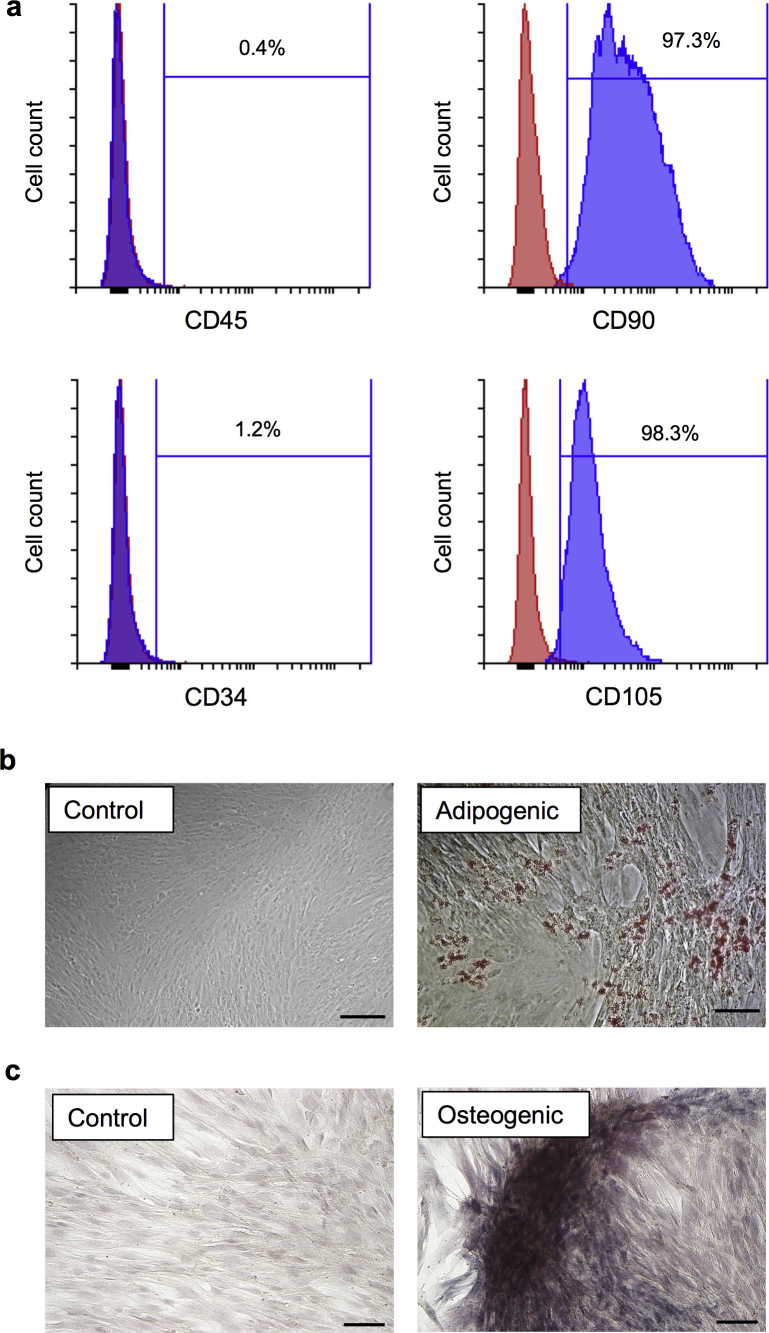
Characterization of human amnion-derived (Ha) MSCs. **(a)**. CD marker expression of human amnion-derived MSCs (passage 12) was assessed by flow cytometric analysis. Red; IgG control, Blue; CD45/90/34/29 staining. **(b,c)**. Differentiation of human amnion-MSCs to adipocyte (b; Oil Red-O staining) and osteocytes (c; Alizarin red staining) was confirmed. Control; cultured in usual medium; Scale bar = 100 μm (b) and 50 μm (c).

**Fig. s3:**
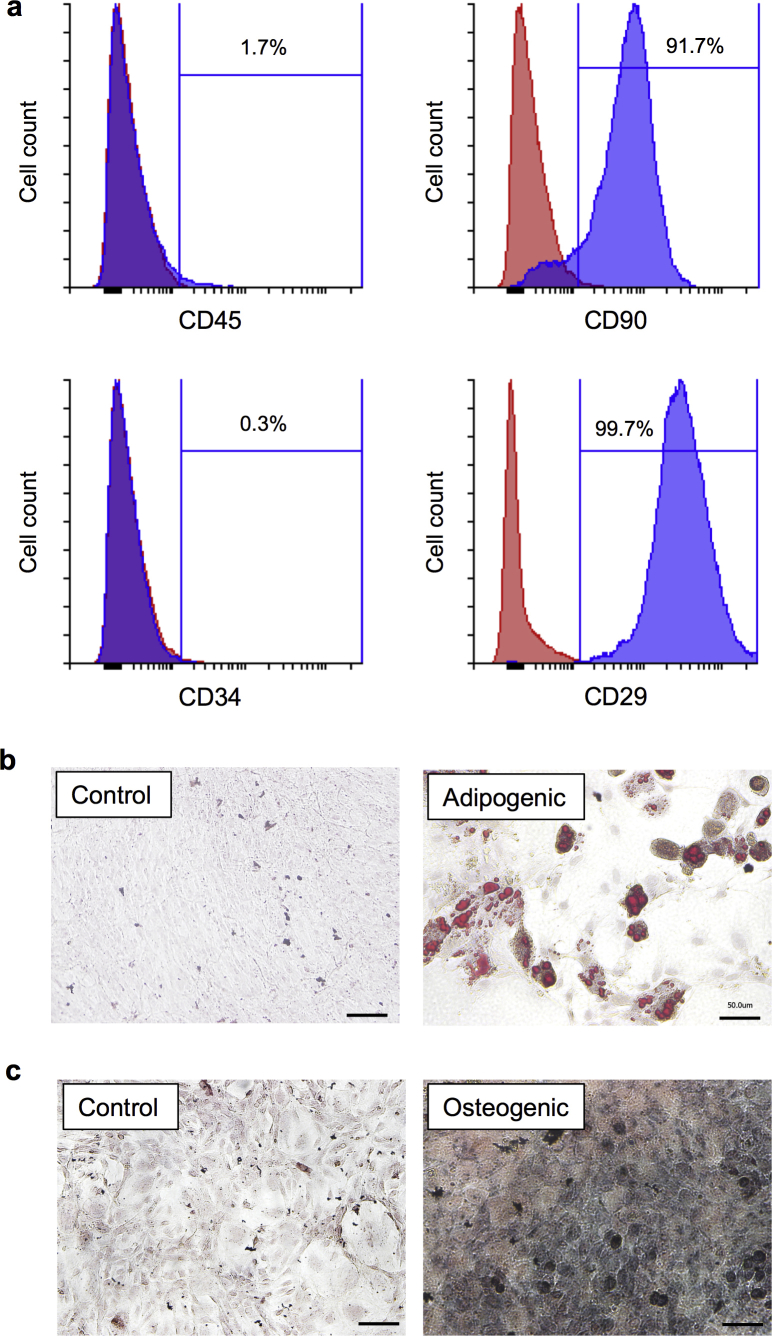
Characterization of rat fetal membrane-derived (Rf) MSCs. (a). CD marker expression of rat fetal membrane-derived MSCs (passage 4) was assessed by flow cytometric analysis. Red; IgG control, Blue; CD45/90/34/29 staining. (b,c). Differentiation of fetal membrane-derived MSCs to adipocyte (b; Oil Red-O staining) and osteocytes (c; Alizarin red staining) was confirmed. Control; cultured in usual medium; Scale bar = 100 μm (b) and 50 μm (c).

**Fig. s4:**
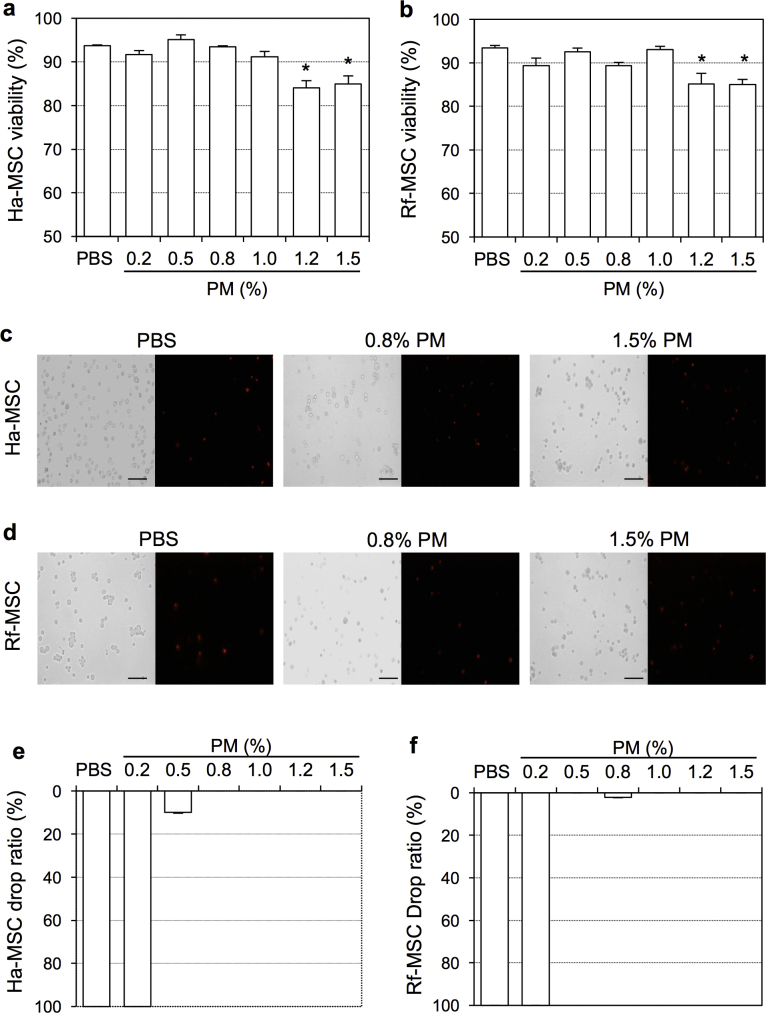
**Optimization the PM-MSC complex using human amnion-derived MSCs and rat fetal membrane-derived MSCs**. (**a, b**). Viability of human amnion-derived MSCs (a) and rat fetal membrane-derived MSCs (b) at 10 min after mixture with different doses of PM *in-vitro* was assessed by propidium iodide staining. Over 1.2% PM showed toxicity to MSCs. n=4 in each point; *p<0.05 vs. corresponding value of the PBS group. Scale bar = 50 μm. (**e, f**). Retention of the PM-MSC complex using human amnion-derived MSCs (e) and rat fetal membrane-derived MSCs (f) in different doses of PM was assessed by a tilting test on the plastic plate *in-vitro*. Over 0.8% PM enabled appropriately retention. *n=4 in each point.*

**Fig. s5:**
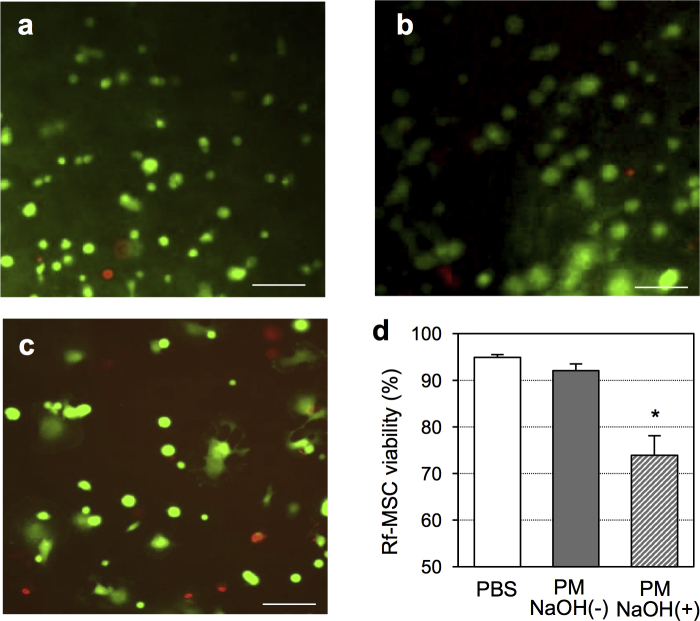
**Viability of MSCs in the PM-MSC complex with neutralization** (Supplement to Fig. 2a and b). Viability of rat fetal membrane-derived MSCs at 10 min after mixture with PM (final dose 0.8%) with or without neutralization using NaOH was assessed by the Live and Dead Cell assay. Representative images in no-PM control (**a**; PBS group), no-neutralization group **(b**; PM NaOH(−) group**)** and NaOH-neutralization group **(c**; PM NaOH(+) group**)** are shown. Green and red fluorescence represent live and dead cells, respectively. Scale bar = 50 μm. The bar graph presents the averaged viability data (**d**). *n=4 in each group*.

**Fig. s6:**
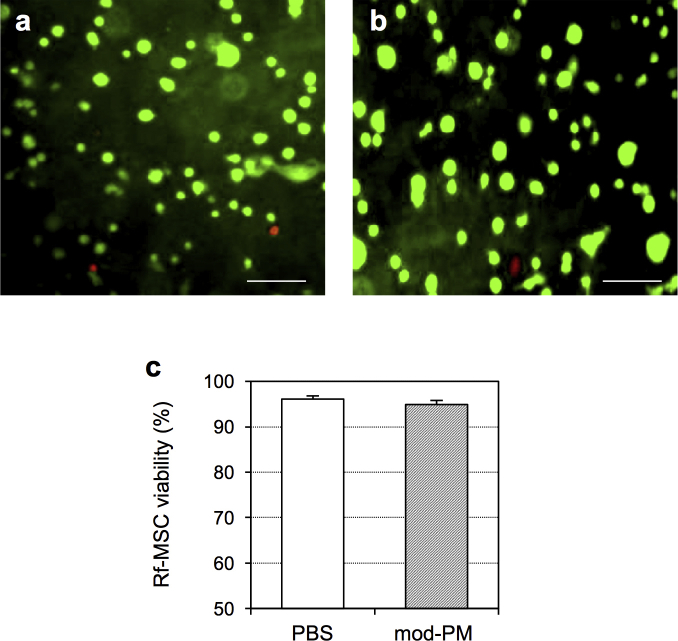
**Viability of MSCs in the modified PM-MSC complex** (Supplement to Fig. 3b and c). Viability of rat fetal membrane-derived MSCs at 10 min after mixture into modified PM (mod-PM; final dose 0.8%) *in-vitro* was assessed by using the Live and Dead Cell assay. Representative pictures in the no-PM control (**a**; PBS group) and modified PM group (b; mod-PM group) are presented. Green and red fluorescence represent live and dead cells, respectively. Scale bar = 50 μm. The bar graph presents the averaged viability of the cells (**c**). *n=4 in each group.*
