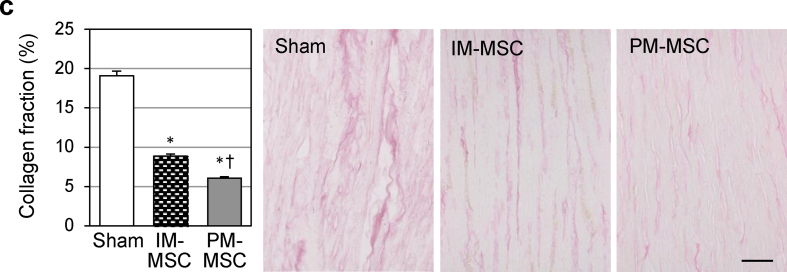# Corrigendum to “Self-assembling peptide hydrogel enables instant epicardial coating of the heart with mesenchymal stromal cells for the treatment of heart failure” [Biomaterials 154 (2018) 12–23]

**DOI:** 10.1016/j.biomaterials.2024.122500

**Published:** 2024-04

**Authors:** Yuki Ichihara, Masahiro Kaneko, Kenichi Yamahara, Marinos Koulouroudias, Nobuhiko Sato, Rakesh Uppal, Kenji Yamazaki, Satoshi Saito, Ken Suzuki

**Affiliations:** a William Harvey Research Institute, Barts and the London School of Medicine and Dentistry, Queen Mary University of London, United Kingdom; b Cardiovascular Surgery, Tokyo Women's Medical University, Japan; c Transfusion Medicine and Cellular Therapy, Hyogo College of Medicine, Japan; d Kaneka Corporation, Osaka, Japan

The authors regret that there were errors in Fig. 7c of this paper (Sham and PM-MSC panels). The corrected version is shown below. This correction does not alter the results, graphs, or conclusions of the study. The authors would like to apologise for any inconvenience caused.

**Figure:**