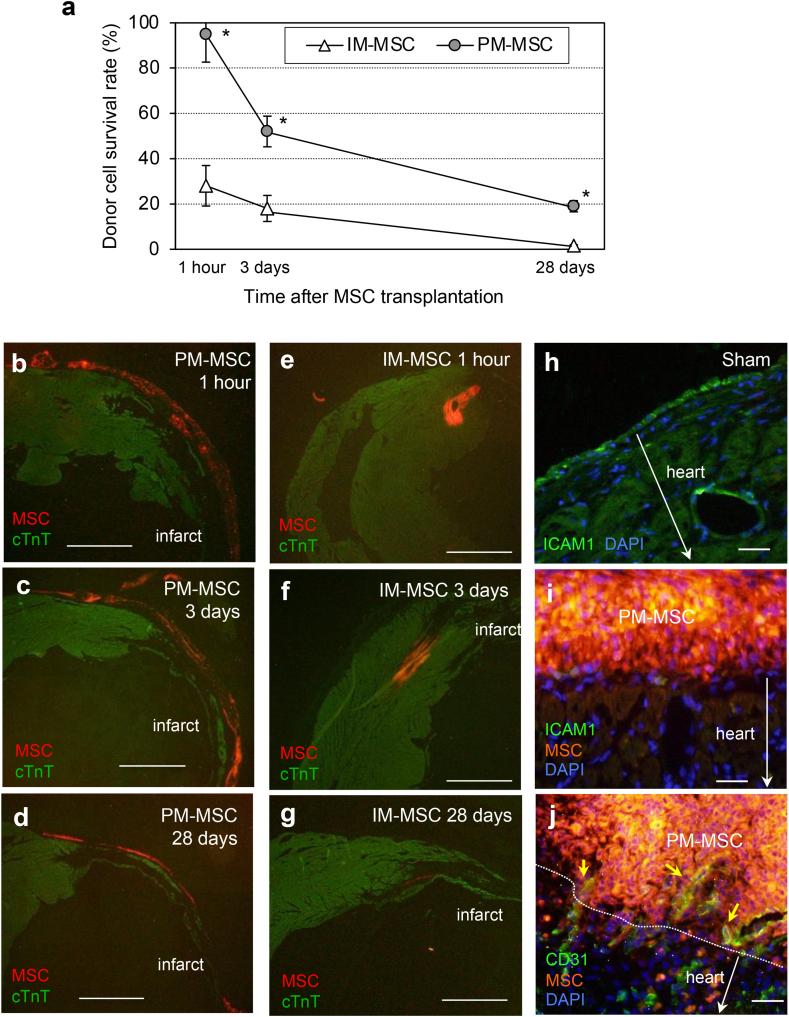# Corrigendum to “Self-assembling peptide hydrogel enables instant epicardial coating of the heart with mesenchymal stromal cells for the treatment of heart failure” [Biomaterials 154 (2018) 12–23]

**DOI:** 10.1016/j.biomaterials.2024.122611

**Published:** 2024-09

**Authors:** Yuki Ichihara, Masahiro Kaneko, Kenichi Yamahara, Marinos Koulouroudias, Nobuhiko Sato, Rakesh Uppal, Kenji Yamazaki, Satoshi Saito, Ken Suzuki

**Affiliations:** a William Harvey Research Institute, Barts and the London School of Medicine and Dentistry, Queen Mary University of London, United Kingdom; b Cardiovascular Surgery, Tokyo Women's Medical University, Japan; c Transfusion Medicine and Cellular Therapy, Hyogo College of Medicine, Japan; d Kaneka Corporation, Osaka, Japan

The authors regret that there were errors in Fig. 6e–g, h, j of this paper. The corrected version of Fig. 6 is shown below. This correction does not alter the conclusion of the study. The authors would like to apologise for any inconvenience caused.

**Figure:**